# A Perfused In Vitro Human iPSC-Derived Blood–Brain Barrier Faithfully Mimics Transferrin Receptor-Mediated Transcytosis of Therapeutic Antibodies

**DOI:** 10.1007/s10571-023-01404-x

**Published:** 2023-09-12

**Authors:** Floriana Burgio, Carine Gaiser, Kevin Brady, Viviana Gatta, Reiner Class, Ramona Schrage, Laura Suter-Dick

**Affiliations:** 1 University of Applied Sciences and Arts Northwestern Switzerland (FHNW), Muttenz, Switzerland; 2 Development Sciences, UCB Biopharma SRL, Braine L’Alleud, Belgium; 3 Neuroscience Therapeutic Area, UCB Biopharma SRL, Braine L’Alleud, Belgium

**Keywords:** Induced pluripotent stem cells-derived brain microvascular endothelial cells, Microfluidic system, Blood–brain barrier, Receptor-mediated transcytosis, Transferrin receptor, Biologics

## Abstract

Delivering biologics to elicit a therapeutic response in the central nervous system (CNS) remains challenging due to the presence of the blood**–**brain barrier (BBB). Receptor-mediated transcytosis is a strategy to improve brain exposure after systemic drug administration. The availability of a clinically relevant in vitro BBB model is crucial to investigate transcytosis pathways and to predict the penetration of biologics into the CNS. We created a perfused human in vitro BBB model made of induced pluripotent stem cells (iPSC)-derived brain microvascular endothelial cells (BMEC) for studying transferrin receptor-mediated transcytosis. iPSC-derived BMEC were seeded in the top channel of a three-lane microfluidic device (OrganoPlate®). After 2 days in culture, the established cell model exhibited relevant BBB features, including physiological transendothelial electrical resistance in a transwell setting (1500 Ω*cm^2^), reduced apparent permeability (Papp) to the fluorescence tracer Lucifer yellow (20-fold less than cell-free chips), expression of key BBB markers such as tight junctions proteins, transporters, receptors and functional P-gp efflux pump. Moreover, the model exhibited functional transferrin receptor-mediated uptake and transcytosis. To assess selective transferrin receptor-mediated transcytosis, a mixture of anti-human transferrin receptor (MEM-189) and control (sheep IgG anti-bovine serum albumin) antibodies was perfused in the top channel for 2 h. The Papp of MEM-189 was 11-fold higher than that of the control antibody, demonstrating facilitated receptor-mediated transcytosis. Compared to published work reporting a 2-fold ratio, this result is remarkable and establishes the suitability of our model for exploring receptor-mediated transcytosis and screening of antibodies for putative brain shuttle application.

**Graphical Abstract:**

A perfused in vitro human model made of iPSC-derived BMEC with the chief characteristics (barrier tightness, functionality) of the human BBB can be applied to study transferrin receptor (TfR)-mediated transcytosis of therapeutic antibodies. This may bring critical advances in drug shuttle technology. Graphical abstract generated with biorender.com.

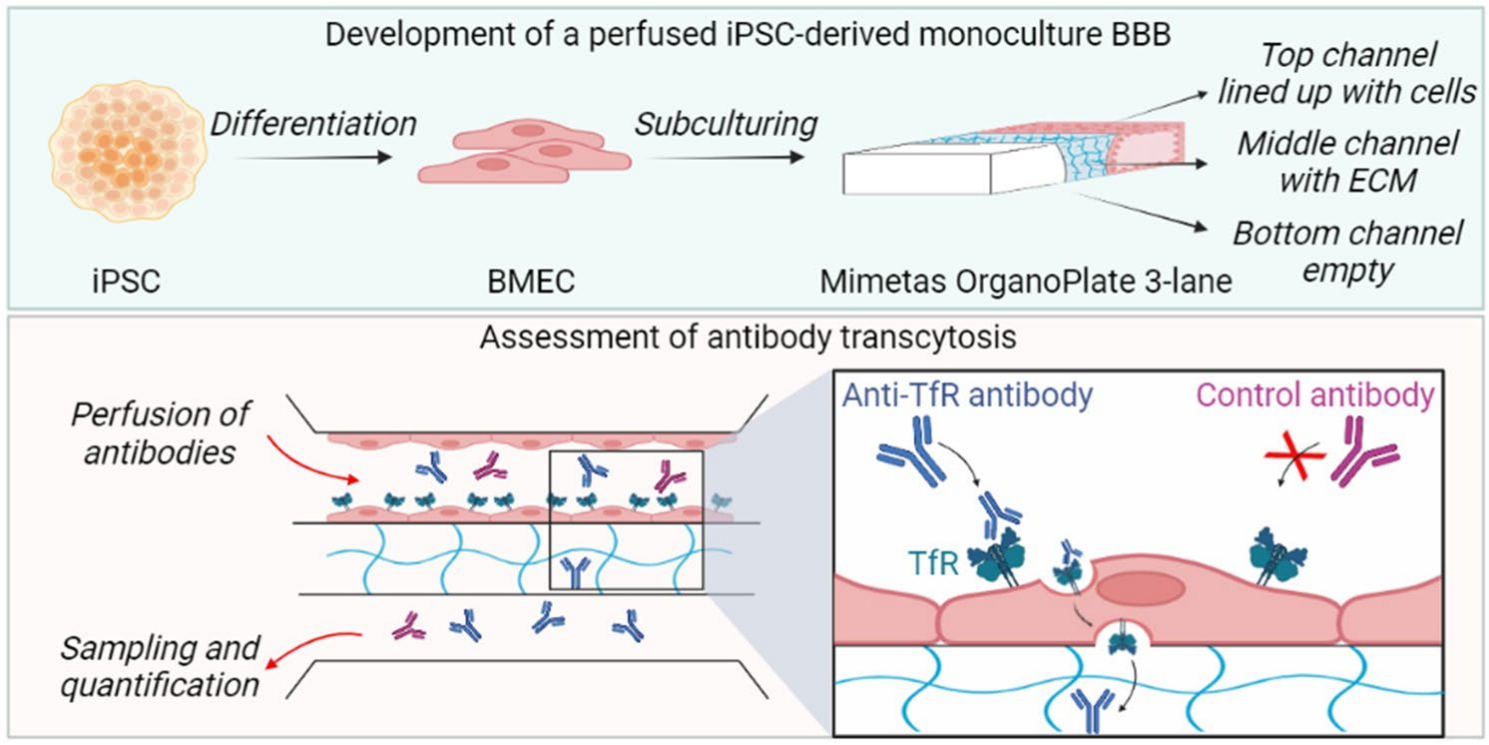

**Supplementary Information:**

The online version contains supplementary material available at 10.1007/s10571-023-01404-x.

## Introduction

The development of effective biologics, such as therapeutic antibodies, for the treatment of neurological disorders requires successful delivery through the blood–brain barrier (BBB). The BBB is composed of several cell types, namely brain microvascular endothelial cells (BMEC), pericytes and astrocytes (Helms et al. 2016). This specialized structure separates the central nervous system (CNS) from the systemic blood circulation, maintaining homeostasis and protecting the brain from potentially harmful substances (Appelt-Menzel et al. 2017). Meanwhile, it selectively prevents biologics from reaching the CNS (Pardridge 2017). The most common method of bypassing the BBB is the injection into the cerebrospinal fluid, but the amount of accumulated biologic may not be sufficient to elicit a therapeutic response (Pardridge 2020; Sadekar et al. 2022). More effective and non-invasive delivery of biologics can be achieved by leveraging specialized receptors on the apical plasma membrane of BMEC. The most appealing option is receptor-mediated transcytosis. Indeed, combining biologics with receptor-mediated transcytosis-targeting antibodies against highly expressed endogenous receptors can result in increased CNS exposure (Niewoehner et al. 2014; Zuchero et al. 2016; Fang et al. 2017; Pardridge 2017; Farrington et al. 2014). Strong evidence supports the use of antibodies against the transferrin receptor as a brain shuttle module to mediate the delivery (Yu et al. 2011; Niewoehner et al. 2014). However, antibody properties strongly influence their interaction with receptors and their intracellular fate, determining how efficiently they can be transcytosed to reach the target site (Yu et al. 2011; Niewoehner et al. 2014; Sade et al. 2014). In vitro BBB models with physiologically relevant features are critical for identifying novel delivery approaches or exploiting already known transcytosis routes. To date, multicellular BBB models have been successfully established in transwell-based static systems (Cecchelli et al. 2014; Appelt-Menzel et al. 2017). In addition, endothelial cells need to be exposed to shear forces from blood circulation for proper barrier formation, making flow-based dynamic systems a natural choice for designing adequate in vitro models (Siddharthan et al. 2007; Cucullo et al. 2011). The combination of microfluidic platforms with ongoing advances in induced pluripotent stem cells (iPSC)-derived BMEC has the potential to result in a highly relevant and predictive BBB model (Fabre et al. 2019). Indeed, iPSC-derived BMEC have shown substantial advantages over primary and immortalized cells as they overcome limitations such as lack of human tissue availability or loss of BMEC-specific properties, respectively. In particular, iPSC represent a stable source displaying characteristics such as scalability, self-renewal, and potential formation of isogenic and personalized BBB models (Canfield et al. 2017). The ability to screen several compounds at once is an additional requirement for the development of a relevant model for drug screening. Several groups have already described the use of the OrganoPlate® 3-lane (Mimetas) to establish in vitro BBB models that could be potentially used in preclinical studies. The system boasts high-throughput readouts and biologically relevant conditions, such as flow, the possibility to implement co-cultures, and the absence of artificial membranes (Koo et al. 2018; Wevers et al. 2018; van Duinen et al. 2020; Fengler et al. 2022; Kurosawa et al. 2022).

In this work, the OrganoPlate® 3-lane was used to create a perfused human in vitro BBB model made of iPSC-derived BMEC for investigation of transferrin receptor-mediated transcytosis. A comprehensive characterization was performed to ensure the development of a BBB model with physiologically relevant barrier features. Demonstration of transferrin receptor-mediated transport was carried out to guarantee its appropriate functionality. To this end, the transcytosis rate of an antibody against the transferrin receptor, MEM-189, and a control antibody were examined. The antibody against the transferrin receptor was able to cross the iPSC-derived BMEC barrier to an 11-fold higher extent than the control antibody. To the best of our knowledge, our findings show for the first time that iPSC-derived BMEC cultured in the Mimetas platform can be used for the evaluation of selective antibody receptor-mediated transcytosis, an essential requirement for potential brain shuttle technology application.

## Methods

### Cell Lines and Cell Culture

The induced pluripotent stem cells (iPSC), SIGi001-A-2 (66540357, EBiSC, England), were cultured in mTeSR^TM^1 culture medium (05850, StemCell Technologies, France) in 6 wells plate pre-coated with 0.333 mg/ml Matrigel Growth Factor Reduced (354230, Corning, Netherlands) for 1 h at 37 °C. The primary human brain vascular pericytes, HBVP (1200, ScienCell Research Laboratories, USA), were cultured in complete pericyte medium (1201, ScienCell Research Laboratories, USA) in flasks pre-coated with 2 μg/cm^2^ poly-l-lysine (P4707, Sigma-Aldrich, Germany). All cells were kept at 37 °C, in a 5% CO_2_ atmosphere. For differentiation of iPSC into iPSC-derived BMEC, a two-step protocol was followed as reported in Katt et al*.* (2016). 300.000/9.5 cm^2^ iPSC were cultured in mTeSR^TM^1. Differentiation was initiated by growing 60–70% confluent colonies with unconditioned medium for 5 or 6 days. The unconditioned medium consisted of DMEM/F12 (12660012, Thermo Fisher, Switzerland) supplemented with 20% KnockOut™ Serum Replacement (10828010, Thermo Fisher, Switzerland), 1% non-essential amino acids (11140050, Thermo Fisher, Switzerland), 0.5% L-glutamine (G7513, Sigma-Aldrich, Germany), and 0.836 μM beta-mercaptoethanol (31350010, Thermo Fisher, Switzerland). Subsequently, the medium was switched to endothelial cell serum-free medium (11111044, Thermo Fisher, Switzerland) supplemented with 1% human platelet-poor derived serum (P2918, Sigma-Aldrich, Germany), 20 ng/ml basic Fibroblast Growth Factor (bFGF; GF003, Sigma-Aldrich, Switzerland), and 10 μM all-trans retinoic acid (RA; R2625, Sigma-Aldrich, Germany). Cells remained in complete endothelial cell medium for 2 days before being subcultured in the system of choice as reported below.

### Characterization of iPSC and iPSC-Derived BMEC in 2D Cell Culture

For cell evaluation in 2D, 20.000/0.33 cm^2^ iPSC and 100.000/0.33 cm^2^ iPSC-derived BMEC were seeded in vessels pre-coated with 0.333 mg/ml Matrigel Growth Factor Reduced for 1 h at 37 °C or with a 50/50 (v/v) mixture of 100 μg/ml collagen IV (C5533, Sigma-Aldrich, Germany) and 50 μg/ml fibronectin (F1056, Sigma-Aldrich, Germany) at 37 °C overnight, respectively. For fluorescence imaging analysis, CELLview™ Slides Greiner Bio-One (7.543.979, Huberlab, Switzerland) were used. Pluripotency of iPSC was evaluated at the gene expression level by using the TaqMan® hPSC Scorecard™ Panel service offered by Life Technologies Corporation. For analysis of pluripotency at the protein level, cells were fixed and stained with the Pluripotent Stem Cell 4-Marker Immunocytochemistry Kit (A24881, Thermo Fisher, Switzerland) according to the manufacturer’s protocol. The ability of iPSC to differentiate into the three germ layers was investigated by using the STEMdiff™ Trilineage Differentiation Kit (05230, Stemcell technologies, France) according to the manufacturer’s protocol. Cells were fixed on day 5 for mesoderm and endoderm or day 7 for ectoderm and labeled with specific lineage primary antibodies (Table 1).

**Table 1:** List of primary antibodies used for three germ layers characterization

Marker	Catalog number	Dilution
Brachyury T (mesoderm)	Mouse anti-human, 14-9770-82, Thermo Fisher	1:100
PAX6 (ectoderm)	Rabbit anti-human, 42-6600, Thermo Fisher	1:125
SOX17 (endoderm)	Mouse anti-human, MA5-24885, Thermo Fisher	1:50

### Establishment of Static- and Dynamic-Based In Vitro BBB Models

For the static-based BBB model, 6.5 mm transwells with 0.4 μm pore polycarbonate membrane inserts (10147291, Fisher Scientific, Switzerland) were used. For the co-culture model, the bottom side of the membrane was pre-coated with 2 μg/cm^2^ poly-l-lysine at 37 °C for 1 h, followed by coating of the top side with a 50/50 (v/v) mixture of 100 μg/ml collagen IV and 60 μg/ml fibronectin at 37 °C overnight. 300.000/0.33 cm^2^ iPSC-derived BMEC and 20.000/0.33 cm^2^ HBVP were seeded on the apical and bottom sides of the membrane, respectively, and co-cultured for 2 days. For the dynamic-based BBB model, the microfluidic 3D cell culture OrganoPlate® 3-lane (4004-400-B, Mimetas, Netherlands) was used. According to the manufacturer’s protocol, 4 mg/ml collagen I-based extracellular matrix (ECM) gel (3447-020-01, R&D systems, Switzerland) was dispensed in the gel inlet of the chip at 37 °C for 1 h. Then, coating of the top channel was performed with a 50/50 (v/v) mixture of 100 μg/ml collagen IV and 60 μg/ml fibronectin at 37 °C overnight. Therefore, 50.000/chip iPSC-derived BMEC were seeded in the top channel and the plate was incubated flat for 5 h before placing it on an interval rocker switching between a + 7° and − 7° inclination every 8 min, allowing a bidirectional flow-induced shear stress of ~ 1.2 dyne/cm^2^ for 2 days (Wevers et al. 2018).

### Assessment of the Barrier Tightness

Transendothelial electrical resistance (TEER) measurement was performed by placing the transwells into a CellZscope system (nanoAnalytics, GmbH) and values were recorded in real-time and analyzed with the CellZscope software.

Permeability was measured after exposure to the following molecules: Lucifer yellow (LY) CH dipotassium salt (L0144, Sigma-Aldrich, Germany) or Alexa Fluor™ 488 IgG (A11078, Thermo Fisher, Switzerland). Under static conditions, 100 µl of 100 µg/ml LY solution was added to the apical chamber and the plate was placed on an orbital shaker at 100 rpm, for 1 h. Under dynamic conditions, 500 µg/ml LY or 200 µg/ml Alexa Fluor™ 488 IgG was perfused in the top channel of the chip according to the manufacturer’s protocol “Barrier Integrity Assay in the OrganoPlate®” for up to 6 h or 4 h, respectively. Fluorescence of the basolateral compartments was read with a FlexStation 3 microplate reader (Molecular Device, LCC) at excitation/emission 428/540 nm for LY, and 495/519 nm for Alexa Fluor™ 488 IgG. Apparent permeability (Papp) was calculated in cm/s according to the following equation: Papp (cm/s) = V_B_/(AC_AO_) × (ΔC_B_/Δ_T_), where V_B_ is the volume in the basolateral chamber (0.6 cm^3^), A is the surface area of the filter (0.33 cm^2^), C_AO_ is the initial concentration in the apical chamber, and ΔC_B_/Δ_T_ is the change of concentration in the basolateral chamber over time. Under dynamic conditions, V_B_ and A were adjusted accordingly (0.04 cm^3^ and 0.005 cm^2^, respectively).

For hyperosmotic BBB opening, iPSC-derived BMEC were pre-incubated with 1.4 M mannitol (M4125, Sigma-Aldrich, Germany) at 37 °C for 15 min followed by perfusion of 500 µg/ml LY solution for up to 6 h. The effect of mannitol on the barrier was monitored by fluorescence imaging with a Zeiss Colibri 7 LED system and by quantification of Papp of LY as reported above.

### Immunocytochemistry Analysis

For immunocytochemistry, cells were fixed with 4% paraformaldehyde for 10 min, permeabilized with 0.1% Triton X-100 for 5 min, and blocked with 3% bovine serum albumin (BSA; A2153, Sigma-Aldrich, Germany) for 1 h at RT. Cells were incubated with primary antibodies overnight at 4 °C, and then with fluorescent secondary antibodies for 60 min at RT (Table 2). Nuclei were counterstained with DAPI for 2 min (2D culture) or 15 min (3D culture) at RT. Pictures were taken with a Zeiss Colibri 7 LED system or with a confocal laser scanning microscope (Olympus, FV3000).

**Table 2:** List of primary antibodies used for immunofluorescence staining

Marker	Catalog number	Dilution
CD31	Rabbit anti-human, ab28364, Abcam	1:50
Claudin5	Rabbit anti-human, ab15106, Abcam	1:100
Glut1	Rabbit anti-human, MA5-31960, Thermo Fisher	1:100
Insulin receptor	Mouse anti-human, MA5-13783, Thermo Fisher	1:20
LDL receptor	Rabbit anti-human, PA5-115504, Thermo Fisher	1:100
Occludin	Mouse anti-human, 33-1500, Thermo Fisher	1:250
P-gp	Mouse anti-human, MA5-13854, Thermo Fisher	1:50
Transferrin receptor	Mouse anti-human, 16-0719-85, Thermo Fisher	1:100
Transferrin receptor	Rabbit anti-human, ab84036, Abcam	1:150
vWF	Mouse anti-human, MA5-14029, Thermo Fisher	1:100
ZO1	Mouse anti-human 33–9100, Thermo Fisher	1:100

### Gene Expression Analysis

For gene expression analysis, total RNA was extracted from at least 5 pooled biological replicates according to the manufacturer’s protocol “TRizol Reagent life technologies™”. RNA was then purified with the RNA cleanup kit (74204, Qiagen, Switzerland) and reverse transcribed to cDNA. Expression was evaluated by qRT-PCR using TaqMan probes (Thermo Fisher, Switzerland) specific for the gene of interest: *ABCB1 (Hs00184500_m1), ABCC1 (Hs00219905_m1), ABCG2 (Hs01053790_m1), SLC2A1 (Hs00892681_m1), TRFC (Hs00951083_m1) and B2M (Hs00187842_m1)* as the housekeeping gene. Results were reported as ΔCt values (Ct gene of interest–Ct housekeeping gene).

### Functional Evaluation of P-glycoprotein and Transferrin Receptor

To assess the activity of the efflux pump, P-glycoprotein (P-gp), iPSC-derived BMEC were pre-incubated with 10 µM inhibitor, Cyclosporin A (CsA; 30024, Sigma-Aldrich, Germany), for 30 min, and subsequently exposed to 10 µM substrate, Rhodamine 123 (R123; 83702, Sigma-Aldrich, Germany), for 1 h with or without the inhibitor. Since CsA was reconstituted in ethanol, the same amount of ethanol was added to the samples without inhibitor as control. Efflux was allowed for 1 h and cells were imaged with a Zeiss Colibri 7 LED system before lysis with 1% Triton X-100 for 15 min. Fluorescence was read with a FlexStation 3 microplate reader at excitation/emission 511/534 nm.

For transferrin uptake assay, iPSC-derived BMEC were pre-incubated with 10 mg/ml unlabeled transferrin (T3309, Sigma-Aldrich, Germany) for 20 min, and subsequently exposed to 500 µg/ml Alexa Fluor™ 488 transferrin (T13342, Thermo Fisher, Switzerland) for 4 h with or without unlabeled transferrin. Fluorescence pictures were taken with a confocal laser scanning microscope.

For transferrin transcytosis assay, iPSC-derived BMEC were exposed to a mixture of 500 µg/ml unlabeled transferrin and 500 µg/ml BSA for 2 h. The amount of transferrin in the bottom channel was quantified by ELISA according to the manufacturer’s protocols (Transferrin ELISA kit, Invitrogen, EHTF, Switzerland). To quantify the amount of BSA, an ELISA assay was set up using the following antibodies: sheep polyclonal, antigen affinity purified (A10-113A, Bethyl, Switzerland) and sheep polyclonal, antigen affinity purified HRP (A10-113P, Bethyl, Switzerland). Data were generated using the SoftMax Pro software, and Papp was calculated as described above.

### Investigation of Antibody Transcytosis

The integrity of the barrier was evaluated by incubating iPSC-derived BMEC with 500 µg/ml Texas Red Dextran (D182, Thermo Fisher, Switzerland) for 1 h according to the manufacturer’s protocol “Barrier Integrity Assay in the OrganoPlate®”. Leakage of Texas Red Dextran from the top to the gel channel was evaluated by qualitative imaging analysis. Chips that did not allow the passage of the dye were considered leak-tight and used for antibody transcytosis. Therefore, a mixture of 1.25 µM mouse anti-human transferrin receptor, MEM-189 (NB500-493, Novus Biological, Switzerland), and 1.25 µM sheep anti-BSA (A10-113, Bethyl, Switzerland) antibodies was perfused in the top channel according to the manufacturer’s protocol “Antibody transcytosis assay in the OrganoPlate®”. Texas Red Dextran was also added to the mixture to monitor the barrier integrity at the end of the incubation time. After 2 h, cell culture medium was collected from the bottom channel, and the amount of antibody was quantified by ELISA according to the instructions of the mouse IgG ELISA kit (ab151276, Abcam, UK) and sheep IgG ELISA kit (ab190546, Abcam, UK).

### Statistical Analysis

Data are presented as median with interquartile or as mean ± SD for at least three chips per condition, unless stated otherwise. Statistics were performed using GraphPad Prism version 8 (San Diego, CA, USA) or Microsoft Excel (Version 2306). Homogeneity of variance was assessed with a two-sample F-test for variances when two groups were analyzed or with Bartlett’s or Brown-Forsythe tests when more groups were analyzed. Normality was assessed with Shapiro–Wilk test.

When normality was met, t test was applied when two groups were analyzed or Welch ANOVA or one-way ANOVA tests were applied when more groups were analyzed. When normality was not met, Mann–Whitney or Wilcoxon matched-pairs signed rank tests were applied when two groups were analyzed or Kruskal–Wallis was applied when more groups were analyzed. Longitudinal data were analyzed with RM one-way ANOVA test.

The adopted test is specified in the caption of each graph. Data were considered significant if *p* ≤ 0.05; exact *p* values and details on the statistical analysis are reported in the supplementary information.

## Results

### iPSC are Pluripotent and Can Generate BMEC

**Fig. 1:**
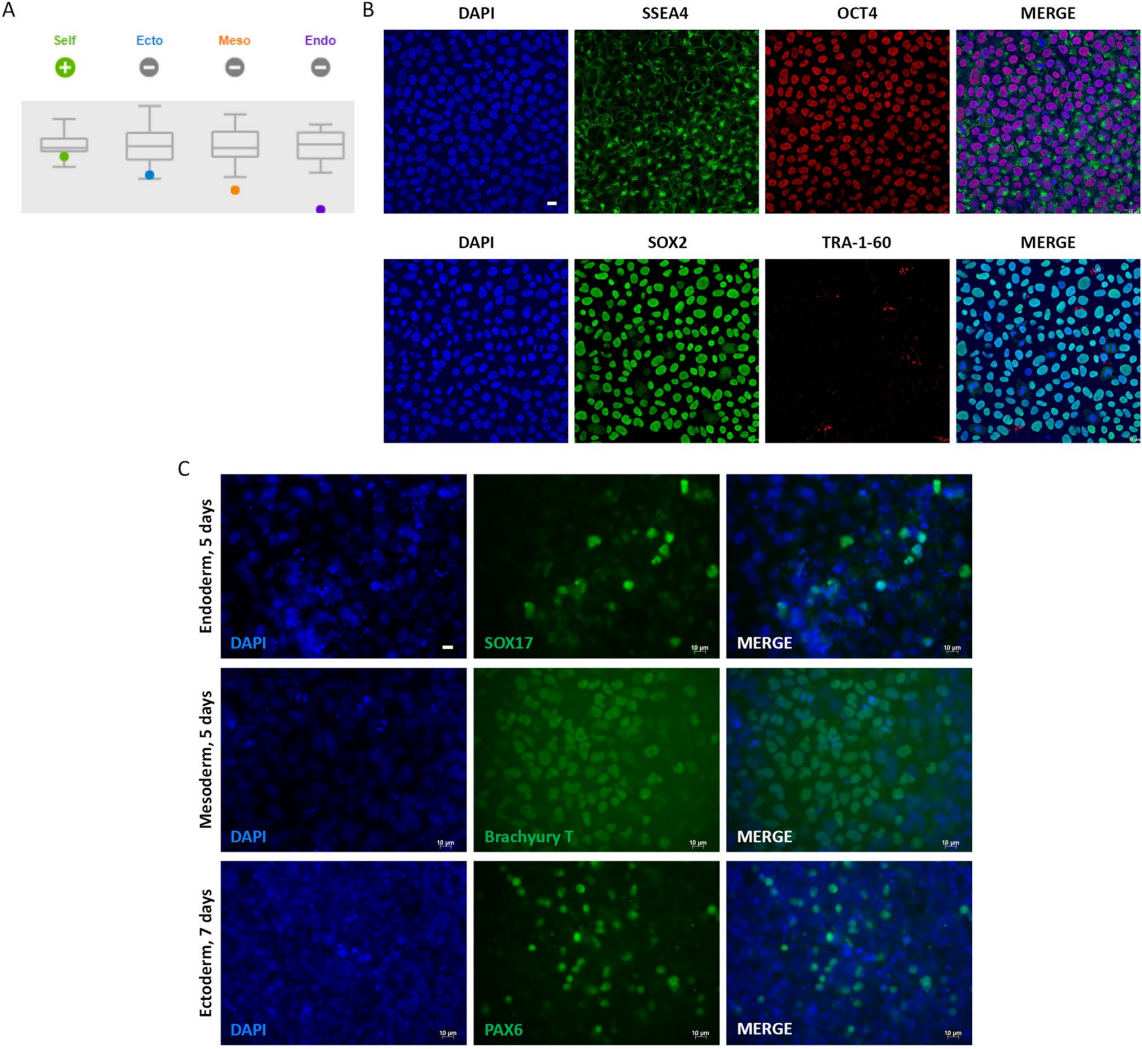
Characterization of iPSC in 2D cell culture. Cell pluripotency was evaluated by **A** TaqMan® hPSC Scorecard and **B** immunostaining of pluripotent stem cell markers OCT4 and TRA-1–60 (red), and SSEA4 and SOX2 (green). DAPI nuclear counter-staining (blue), scale bar: 15 µm. **C** Trilineage differentiation was confirmed by immunostaining of SOX17, Brachyury T, and PAX6 (green) specific for endoderm, mesoderm, and ectoderm. DAPI nuclear counter-staining (blue), scale bar: 10 µm

The human iPSC, SIGi001-A-2, were characterized before establishing the BBB model. TaqMan® hPSC Scorecard™ analysis was performed to assess pluripotency by comparing the gene expression profile of SIGi001-A-2 to the reference set of nine undifferentiated iPSC. According to the analysis software, the cells tested positive for self-renewal markers (Fig. 1A). The expression of key pluripotency proteins OCT4, SSEA4, TRA-1-60 and SOX2 was also confirmed (Fig. 1B). Furthermore, differentiation into the three germ layers was induced and confirmed by the detection of markers SOX17, Brachyury T, and PAX6, which are specific for endoderm, mesoderm, and ectoderm lineages, respectively (Fig. 1C).

Under specific culture conditions, iPSC could be differentiated into BMEC. The BMEC differentiation protocol described in Material and Methods was adapted from Katt et al*.* (2016). The expression of BBB and endothelial markers was investigated in iPSC-derived BMEC (referred to as BMEC) and undifferentiated iPSC, used as control, by immunostaining. Surprisingly, both cell types expressed ZO1, Occludin as well as transferrin receptor and P-gp. However, P-gp and transferrin receptor were relocated to the cell membrane of differentiated BMEC when compared to undifferentiated iPSC that displayed a more diffuse expression pattern. The same pattern was observed for CD31, albeit its expression was weaker and less distinct at the cell membrane (Fig. S1A and B). As further evidence of the differentiation process, BMEC did not express any of the tested pluripotency markers OCT4, TRA-1-60, and SOX2 (Fig. S1C). SSEA4 expression was low and not localized at the cell membrane.

### iPSC Differentiate into Endothelial Cells with BBB Phenotype and Produce a Tight Barrier Under Dynamic Conditions

**Fig. 2:**
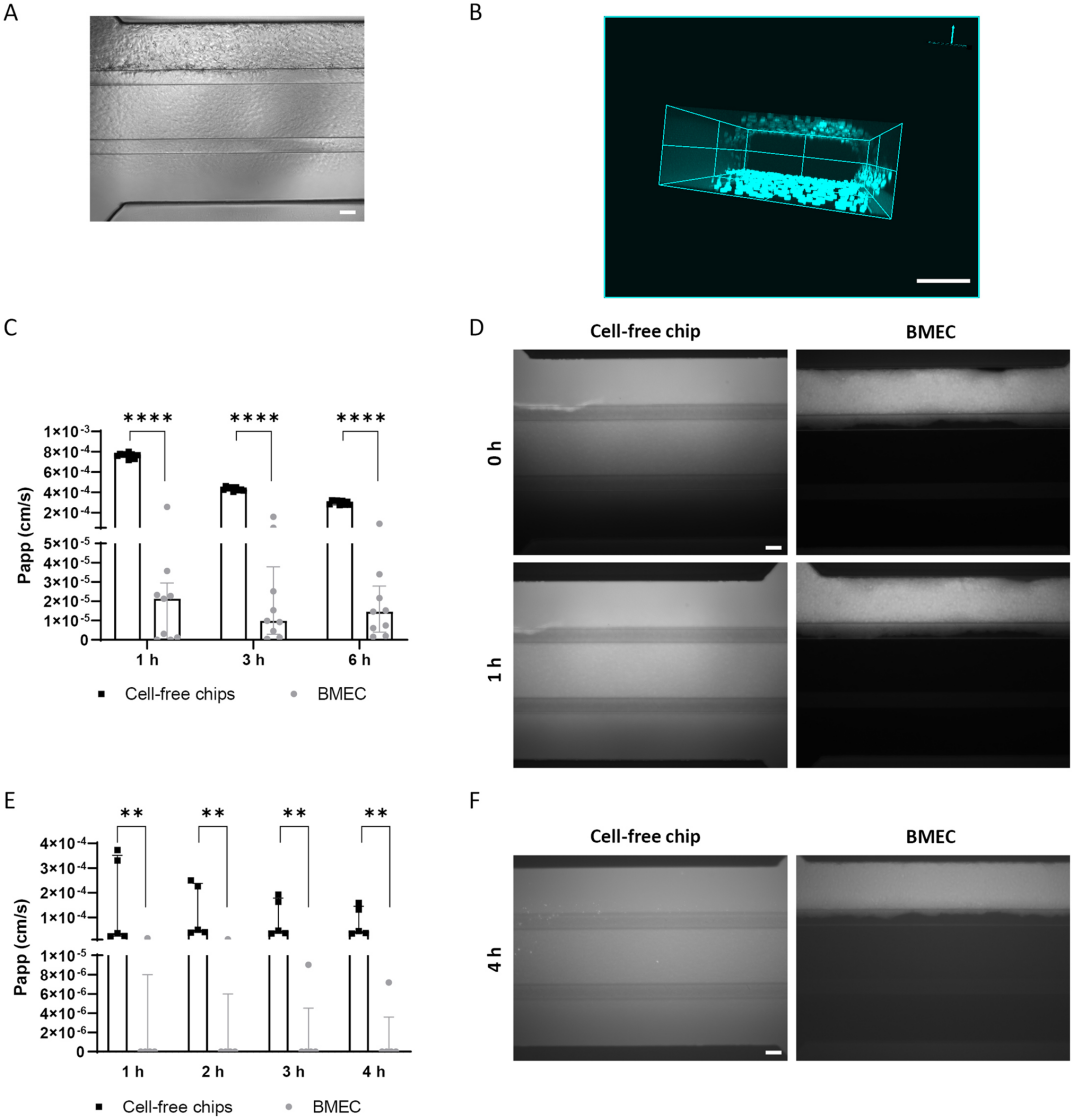
Tightness evaluation of the dynamic-based BBB model. 50.000/chip BMEC were grown in the top channel for 2 days. **A** Brightfield picture showing the cell distribution, scale bar 100 µm. **B** 3D reconstruction of the tubular structure. DAPI nuclear counter-staining in blue, scale bar 100 µm. **C** Papp of 500 µg/ml LY over 6 h, expressed as median with interquartile range (*n* = 9 chips per condition). The statistical analysis was performed using Mann–Whitney test, ****p < 0.0001. **D** Representative fluorescence images of LY solution immediately after perfusion and after 1 h, scale bar: 100 µm. **E** Papp of 200 µg/ml Alexa Fluor™ 488 IgG over 4 h, expressed as median with interquartile range (*n* = 5 chips per condition). The statistical analysis was performed using Mann–Whitney test, **p < 0.01. **F** Representative fluorescence images of Alexa Fluor™ 488 IgG solution after 4 h perfusion, scale bar: 100 µm

**Fig. 3:**
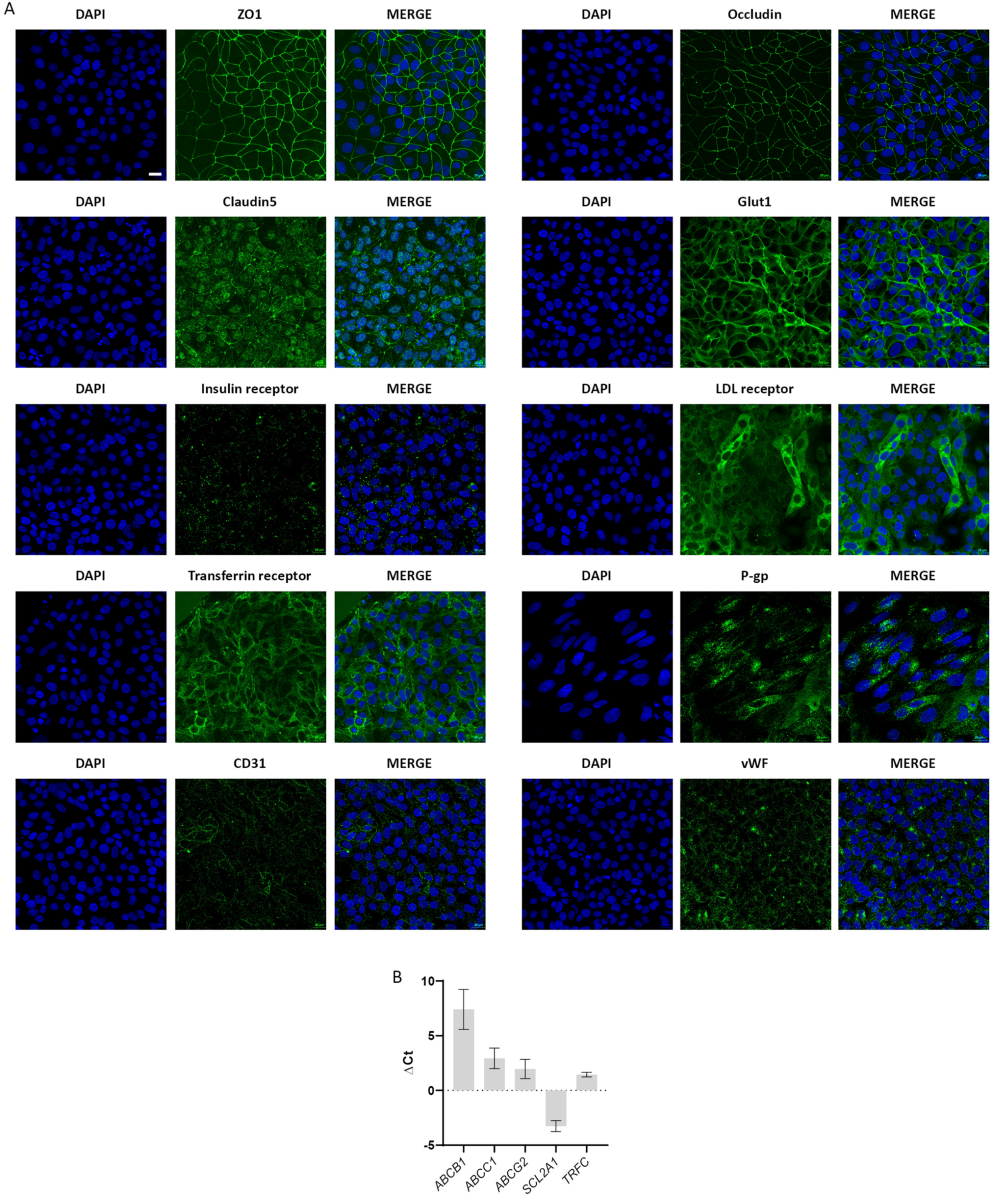
Expression of BBB and endothelial markers in the dynamic-based BBB model. On day 2, cell differentiation was assessed through the expression of BBB and endothelial markers. **A** Immunostaining of ZO1, Occludin, Claudin5, Glut1, insulin receptor, LDL receptor, transferrin receptor, vWF, and CD31 (green). DAPI nuclear counter-staining (blue), scale bar: 20 µm. **B** Gene expression analysis of transporters (*ABCB1*, *ABCC1*, *ABCG2,* and *SCL2A1*) and receptor *(TRFC)*, expressed as mean ± SD (*n* = 3). Values are expressed as ΔCt (Ct gene of interest–Ct housekeeping gene), and *B2M* was used as housekeeping gene

The ability of BMEC to form a barrier was first tested under static conditions in a transwell-based system. Primary HBVP were also included in the model to assess their impact on barrier formation. Furthermore, the withdrawal of bFGF and RA from the medium one day after subculture was considered because it has been reported to maximize the TEER (Stebbins et al. 2016). Therefore, BMEC (300.000/0.33 cm^2^) and HBVP (20.000/0.33 cm^2^) were seeded on the apical and bottom sides of the membrane, respectively. After 2 days of subculture, the BMEC monolayer had a TEER value of 1500 Ω*cm^2^ (Fig. S2A). Papp of LY (100 µg/ml) from the apical to the basolateral direction was up to 6-fold lower in the presence of BMEC than in cell-free transwells (Fig. S2B). The presence of pericytes, as well as the withdrawal of bFGF and RA, did not improve the tightness of the barrier. Consequently, only BMEC were used to develop the model in the OrganoPlate® 3-lane under dynamic conditions. Here, 50.000/chip BMEC were subcultured in the top channel for 2 days to generate a cellular barrier. Brightfield analysis revealed a uniform cell distribution within the lane (Fig. 2A). Furthermore, 3D reconstruction showed a defined tubular structure (Fig. 2B). The permeability of the barrier to LY (500 µg/ml) and Alexa Fluor™ 488 IgG (200 µg/ml) was evaluated over time. After 1 h incubation, Papp of LY and Alexa Fluor™ 488 IgG was ~ 20 and 60-fold lower in the presence of BMEC than in the cell-free chips, respectively (Fig. 2C and E). Fluorescence imaging was also used to demonstrate the tightness of the barrier to the fluorescent molecules (Fig. 2D and F). The expression of the main BBB markers, tight junction proteins (Occludin and Claudin5), tight junction-associated protein (ZO1), transporters (Glut1 and P-gp), receptors (insulin-, LDL-, and transferrin receptor), and endothelial markers (CD31 and vWF) was proven by immunostaining (Fig. 3A). High expression of Glut1 (*SCL2A1*) was also confirmed by qRT-PCR analysis, while efflux pumps, MRP1 and BCRP (*ABCC1* and *ABCG2*), and transferrin receptor (*TRFC*) were expressed at levels slightly lower than the housekeeping gene (*B2M*). The gene expression of P-gp (*ABCB1*) was low, but detectable (Fig. 3B).

### Mannitol Induces Heterogeneous and Transient Disruption of the iPSC-derived BBB

**Fig. 4:**
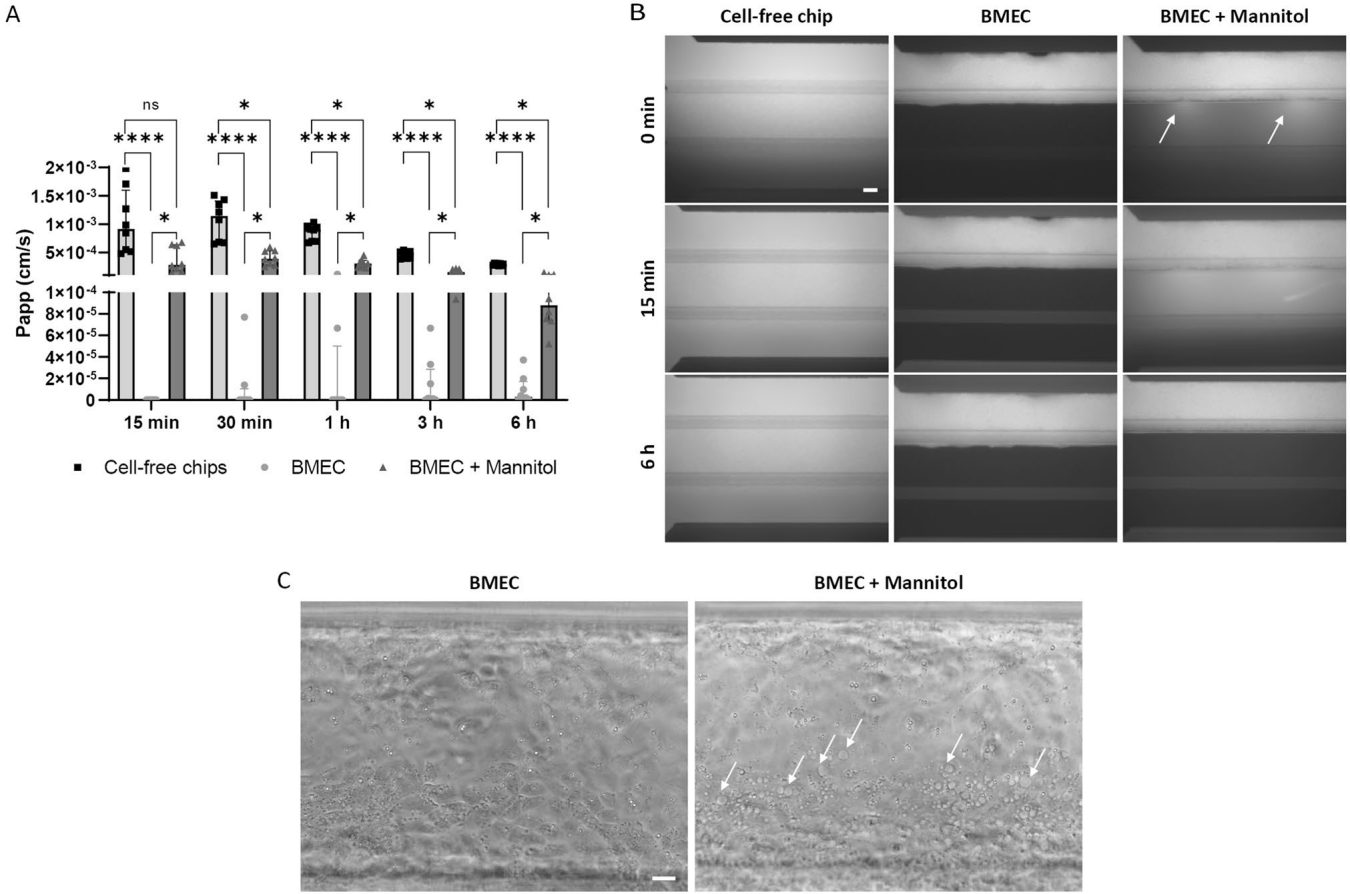
Mannitol-induced BBB opening. To induce transient barrier opening, BMEC were pre-incubated with 1.4 M mannitol. **A** Papp of 500 µg/ml LY over 6 h, expressed as median with interquartile range (*n* = 8 chips per condition). The statistical analysis was performed using Kruskal–Wallis test, Dunn’s multiple comparisons test, *p < 0.05, ****p < 0.0001. **B** Representative fluorescence images of chips perfused with LY solution after 0 min, 15 min, and 6 h. Arrows indicate the focal leaks, scale bar: 100 µm. **C** Vacuolation induced by mannitol. Representative brightfield images taken 30 min after washing of mannitol. Arrows indicate the vacuoles, scale bar: 50 µm

To further investigate the physiological functionality of the barrier, the hyperosmotic agent mannitol was used to induce transient opening of the BBB. Therefore, BMEC were pre-incubated with mannitol (1.4 M) for 15 min before being perfused with LY (500 µg/ml). During this process, brightfield or fluorescence microscopy was used to monitor the status of the endothelium and to visualize the passage of LY. The amount of LY in the bottom channel was quantified over 6 h (Fig. 4A). After 15 min, no LY passed through the untreated BMEC, used as control, whereas increased permeability was measured in the mannitol-treated BMEC. However, the cell layer was only punctually disrupted leading to localized leakiness corresponding to a Papp that was higher than in intact chips and 3-fold lower than in the cell-free chips, after 30 min. This is in line with the fluorescence imaging showing the formation of focal openings along the length of the monolayer (Fig. 4B). After 6 h, no fluorescence was observed in the gel or bottom channel, indicating that the effect was transient. In addition to the functional changes, exposure of BMEC to mannitol also caused morphological changes including BMEC vacuolation (Fig. 4C).

### P-gp and Transferrin Receptor are Functional in iPSC-derived BBB

As a measure of the functionality of iPSC-derived BMEC, the activity of P-gp was assessed both in 2D and 3D cell cultures. BMEC were incubated with 10 µM R123, a known substrate of P-gp. When the efflux through P-gp was blocked with 10 µM CsA, intracellular accumulation of R123 increased. A statistically significant increment was detected in 2D but not in 3D (Fig. S3A and C). This might be ascribed to technical limitations of fluorescence quantification in the microfluidic device. However, the higher intracellular amount of R123 upon inhibition was clearly observed by imaging both in 2D and 3D cell cultures (Fig. S3B and D).

**Fig. 5:**
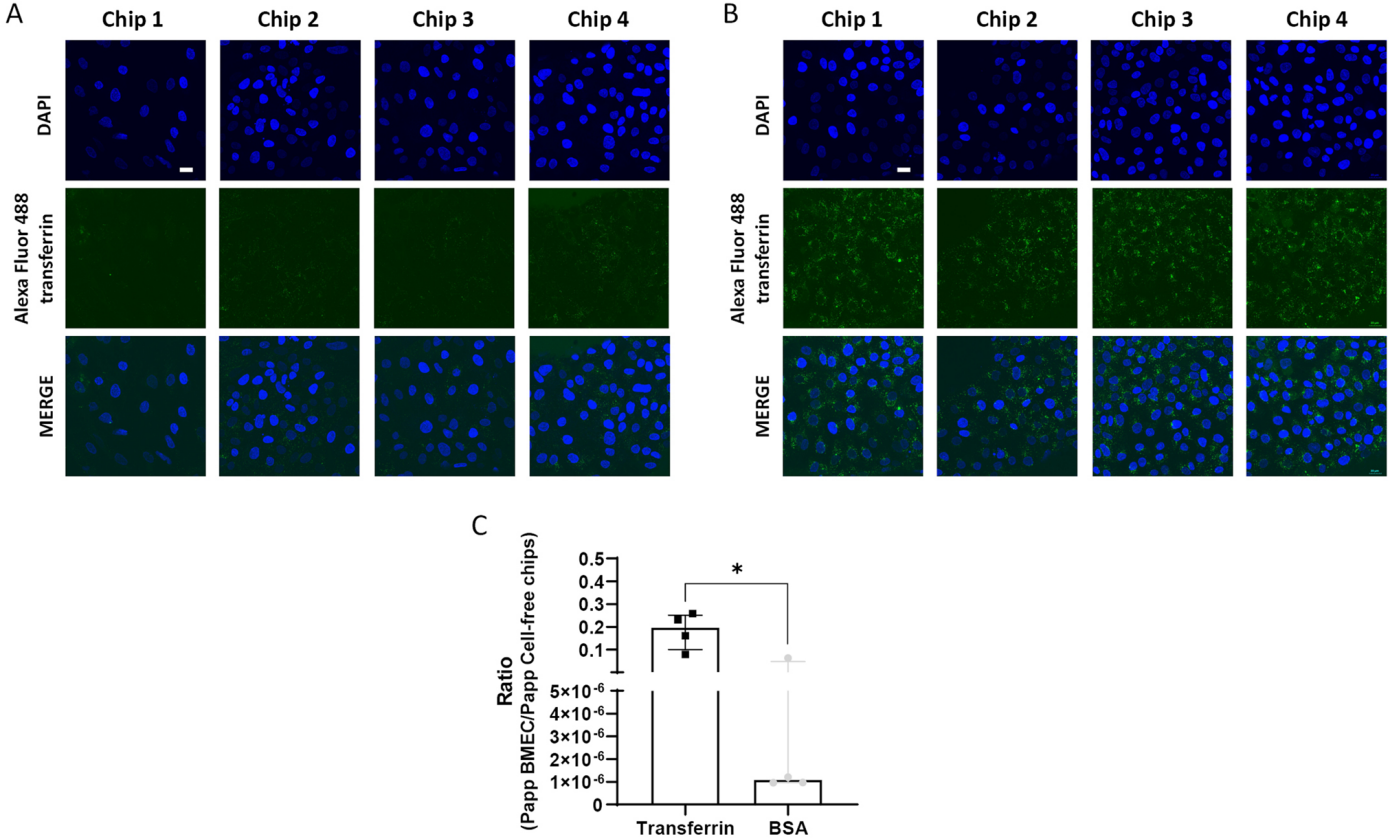
Transferrin receptor-mediated transport. To evaluate transferrin uptake, BMEC were incubated with 500 µg/ml Alexa Fluor™ 488 transferrin for 4 h with (**A**) and without (**B**) 20-fold higher concentration of unlabeled transferrin. DAPI nuclear counter-staining (blue), scale bar: 20 µm. Representative images are shown. **C** To evaluate transferrin transcytosis, BMEC were incubated with a mixture of 500 µg/ml unlabeled transferrin and BSA for 2 h. The concentration of transferrin and BSA in the bottom channel was quantified by ELISA. Papp was calculated accordingly and data are reported as ratio of Papp between BMEC and cell-free chips, expressed as median with interquartile range (*n* = 4 chips per condition). The statistical analysis was performed using Mann–Whitney test, *p < 0.05

Due to its role in receptor-mediated transcytosis, the transferrin receptor-mediated transport was also investigated. Decreased uptake of Alexa Fluor™ 488 transferrin was observed after 4 h competition with 20-fold higher concentration of unlabeled transferrin supporting that the uptake was specifically mediated by the transferrin receptor (Fig. 5A and B). In addition, cells were incubated with a mixture of unlabeled transferrin (500 µg/ml) and BSA (500 µg/ml) to evaluate the specific transcytosis of transferrin. After 2 h incubation, both molecules freely diffused through the gel in the cell-free chips (Fig. S4). The cells hindered the passage of the proteins, as Papp values for transferrin and BSA dropped by 5 and 65-fold in the presence of BMEC, respectively. Moreover, the ratio (Papp BMEC/Papp cell-free chips) of transferrin was 11-fold higher than that of BSA confirming specific receptor-mediated transcytosis (Fig. 5C).

### Transferrin Receptor Promotes Selective Antibody Transcytosis

**Fig. 6:**
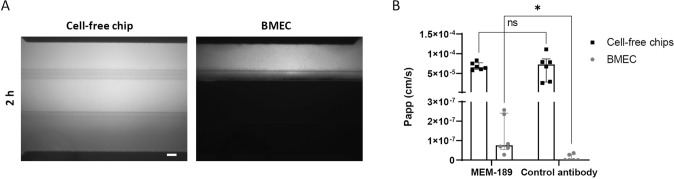
Antibody transcytosis through the dynamic-based BBB model. To evaluate the passage of antibody across the model, a solution made of 1.25 µM MEM-189 (against the transferrin receptor) and control antibody (sheep IgG against BSA), and 500 µg/ml Texas Red Dextran was perfused in the top channel for 2 h. **A** Fluorescence imaging of Texas Red Dextran solution after 2 h perfusion, scale bar: 100 µm. **B** The amount of antibody in the bottom channel was quantified by ELISA and Papp was calculated accordingly, expressed as median with interquartile range (*n* = 6 chips per condition). The statistical analysis was performed using Wilcoxon matched-pairs signed rank test when comparison refers to BMEC or paired t test when comparison refers to cell-free chips, *p < 0.05

To assess the ability of the developed BBB model to promote selective antibody transcytosis, an antibody against the transferrin receptor, MEM-189, was used. A sheep IgG with comparable size against BSA was used as control antibody because it is not expected to bind to any target. Based on qualitative fluorescence imaging, 40 kDa Texas Red Dextran leak-tight chips were selected for the assay. A solution of MEM-189 (1.25 µM) and control antibody (1.25 µM) was infused in the top channel for 2 h. Texas Red Dextran was also added to the solution to ensure the integrity of the chips at the end of the incubation (Fig. 6A). In the cell-free chips, Papp of diffused antibody was comparable. However, in the chips with BMEC, Papp of MEM-189 was 11-fold higher than that of the control antibody validating selective transcytosis (Fig. 6B).

## Discussion

In this work, we established and characterized a perfused in vitro human iPSC-derived BBB model suitable for the investigation of antibody transcytosis and, thus, for studying brain shuttle technology. Targeting BBB receptors, such as transferrin receptor, has the potential to facilitate the transport of large molecules into the brain where they can display their therapeutic effect. Organ-on-a-chip microfluidic devices represent a useful tool for elucidating the interactions of the BBB with potential drugs because they faithfully reproduce the physiological environment and mechanical forces that cells experience in the human body (Brown et al. 2015; Fabre et al. 2019; Fengler et al. 2022). Furthermore, iPSC-derived cells have shown promising in vivo-like characteristics providing information of greater clinical relevance than immortalized or primary cells (Lippmann et al. 2012; Ohshima et al. 2019).

Following these significant advances, we differentiated iPSC into iPSC-derived BMEC and cultured them within the OrganoPlate® 3-lane. Unlike other microfluidic systems, in this device, cells experience bidirectional flow-induced shear stress of ~ 1.2 dyne/cm^2^ and are not in contact with an artificial membrane, but with physiologically relevant extracellular matrix proteins (Wevers et al. 2018). The system is compatible with automated fluid handling and allows the evaluation of 40 chips simultaneously, which is valuable for medium throughput drug screening (Fengler et al. 2022). For drug discovery systems, the choice of cells and their specific characteristics are key elements for the successful implementation of novel models. To ensure the appropriate performance of the undifferentiated cell population, the human iPSC, SIGI001-A-2, were thoroughly characterized by assessing the expression of pluripotency markers and their ability to differentiate into cells of the three germ layers. The differentiation of the iPSC into BMEC was achieved following the protocol described by Katt et al*.* (2016).

The primary goal of this study was to establish a dynamic-based BBB model, but transwells were used to evaluate the ability of iPSC-derived BMEC to form a tight barrier. Several studies have highlighted the role of other cell types in the formation of the BBB (Cecchelli et al. 2014; Lippmann et al. 2015; Canfield et al. 2017; Appelt-Menzel et al. 2017). Indeed, while endothelial cells build the barrier with their specialized features, pericytes, astrocytes, and neurons contribute to the proper development and maintenance of the BBB. In our model, iPSC-derived BMEC monolayer reached physiological TEER (1500 Ω*cm^2^) on day 2 (Fig. S2A), whereas a longer culture time resulted in lower values (Fig. S2C) comparable to what was published (Katt et al. 2016; Stebbins et al. 2016). In our hands, the co-culture with pericytes did not improve barrier tightness. These results strengthen the hypothesis postulated by Jamieson et al*.* that pericytes are not crucial for establishing barrier function in healthy iPSC-derived BMEC monolayer but they might help rescue stressed monolayer through the secretion of soluble factors (Jamieson et al. 2019). Considering the comparable performance and keeping in mind the need for a simple system for screening, we considered a monoculture iPSC-derived BBB model the most suitable system for further experiments under dynamic conditions. Thus, the top channel of the microfluidic device was lined with iPSC-derived BMEC cultured against a collagen I-based ECM gel. Coating the top channel with fibronectin/collagen IV was crucial to ensure proper cell distribution and adhesion. The expression of the tight junction proteins (Occludin and Claudin5), tight junctions-associated protein (ZO1), transporters (P-gp, Glut1, BCRP, and MRP1), receptors (insulin-, LDL-, and transferrin receptor), and endothelial markers (CD31 and vWF) confirmed the successful differentiation of iPSC into endothelial cells with BBB phenotype. After 1 h, Papp values to two different sized fluorescent probes, LY (~ 500 Da) and Alexa Fluor™ 488 IgG (150 kDa), revealed that the generated tubular structures were leak-tight exerting 20 and 60-fold more restriction (*p* < 0.0001 and *p* = 0.0079), respectively, to diffusion of the molecules than cell-free chips. Notably, the tightness of the barrier was maintained for up to 6 h consistently to data previously published (Kurosawa et al. 2022). As further evidence of a functional BBB in the chip, the action of a 15 min mannitol infusion was investigated. The use of hyperosmotic agents, such as mannitol, is often reported as an alternative procedure to mediate drug delivery since it induces the transient opening of the BBB and therefore its increased permeability (Chu et al. 2022). In our system, mannitol caused loosening of the barrier as shown by penetration of LY through the endothelium already 15 min after its perfusion. After 30 min, Papp of LY in mannitol-treated cells was 35-fold higher than in untreated cells (*p* = 0.0452) but still 3-fold lower than in cell-free chips (*p* = 0.0452). This showed that hyperosmotic stress did not completely disrupt the tight junctions network but induced focal leaks, followed by the formation of vacuoles as a consequence of intracellular water loss (Linville et al. 2019, 2020). Within 6 h, the barrier tightness was restored corroborating the transient activity of mannitol in accordance with published data (Linville et al. 2020).

The BBB gatekeeper activity of P-gp is one of the reasons for low drug concentration in the CNS. This transporter is an ATP-dependent efflux pump expressed on the apical side of BMEC that controls the active back-transport of large molecules to the blood (Schinkel 1999). Despite low gene expression, P-gp was detected at the protein level and its activity was confirmed by an increased accumulation of R123 in the cells when P-gp was inhibited with CsA. These results show the presence of  a functional P-gp and are consistent with other reports (Lippmann et al. 2012; Canfield et al. 2017; Neal et al. 2019).

The brain shuttle technology is based on re-engineering biologics of interest by fusing them to peptides or antibodies that allow the crossing of the BBB via receptor-mediated transcytosis and thus act as Trojan horses (Niewoehner et al. 2014; Fang et al. 2017; Pardridge 2017). Because of its high expression on the BMEC surface, the transferrin receptor has been recognized as a good candidate for providing a molecular lift to enhance antibody transcytosis (Yu et al. 2011; Niewoehner et al. 2014). Hence, special emphasis was placed on the characterization of transferrin receptor to assess the suitability of our system for testing potential brain shuttles. Strong cellular uptake of labeled transferrin by iPSC-derived BMEC was evident, indicating functional receptor-mediated endocytosis. However, endocytosis is necessary but not sufficient to lead to transcytosis. Therefore, the passage of transferrin and BSA through our model was assessed. Transferrin is expected to be able to cross the BBB due to the specific binding to its receptor, while BSA is not expected to cross the cellular barrier. Both proteins were able to diffuse to the bottom channel in the absence of cells. The higher diffusion of BSA compared to transferrin across cell-free chips might be ascribed to its smaller molecular weight. A strong difference in the diffusion of these proteins was noted when the chips were lined with iPSC-derived BMEC. The cells reduced the Papp of BSA and transferrin by 65 and 5-fold, respectively, confirming the strict paracellular tightness of our model to proteins. Furthermore, the ratio of Papp between BMEC and cell-free chips of transferrin was 11-fold higher than that of BSA (*p* = 0.0286). These results suggest that proteins can cross the iPSC-derived BMEC barrier mainly via transferrin receptor-mediated transcytosis, a key requirement for an in vitro BBB model. Given the proven functionality of the transferrin receptor enabling transcytosis, the passage of the MEM-189, antibody against the transferrin receptor, and a control antibody, sheep IgG against BSA, was calculated. MEM-189 was chosen because its passage through the BBB has been already demonstrated (Sade et al. 2014; Wevers et al. 2018). Previous studies by Sade et al. showed that MEM-189 binds to the transferrin receptor in a pH-dependent manner, which causes its release from the transferrin receptor in the acidic endosomal environment promoting transcytosis (Sade et al. 2014). In our model, we could demonstrate the ability of MEM-189 to cross a tight iPSC-derived BMEC barrier, as demonstrated by its much higher Papp compared to that of the control antibody of similar structure and molecular weight. These findings are in line with those reported by Wevers et al. (2018). Furthermore, the difference in transcytosis between the two antibodies consistently observed in our study was remarkable. Indeed, the Papp of MEM-189 was 11-fold higher than that of the control antibody (*p* = 0.0313), demonstrating that this BBB model exhibits excellent barrier properties hindering the passage of untargeted antibodies, and a functional transferrin receptor enabling the targeted shuttling of the anti-transferrin receptor antibody, MEM-189. These data clearly demonstrate the superior performance of this iPSC-derived BMEC model in comparison with previously reported systems that showed only a small difference (2-fold) in transcytosis between MEM-189 and a control antibody, despite the use of more complex cellular systems incorporating additional cell types such as pericytes and astrocytes (Wevers et al. 2018). This outcome further substantiates our observation that an in vitro BBB model suitable for the determination of barrier crossing ability of substances can be generated using iPSC-derived BMEC. From a compound screening perspective, this is a major asset, as simpler models based on one adequate cell type rather than co-culture of several cell types of different origins are easier to handle, cheaper, and generally more robust than more complex systems. The relatively low absolute amount of MEM-189 able to cross the iPSC-derived BMEC model is in agreement with previous experiments and may be due to its inherent limited transport across the BBB; it has been described that 40% of MEM-189 is recycled and 20% is retained in the endothelial cells (Sade et al. 2014).

Overall, our model displays appropriate characteristics of the human BBB in terms of barrier tightness and functionality. Although other cell types, such as pericytes and astrocytes, are known to play a critical role in the formation of the neurovascular unit in vivo, our findings suggest that a suitable in vitro BBB model can be achieved with a monoculture of endothelial cells.

Our results clearly demonstrate that this in vitro BBB model can be applied for the investigation of receptor-mediated transcytosis and screening of antibodies for putative brain shuttle targeting transferrin receptor.

### Supplementary Information

Below is the link to the electronic supplementary material.

Supplementary file1 (DOCX 3380 KB)

## Data Availability

The datasets used and/or analyzed during the current study are available from the corresponding author on reasonable request.

## Abbreviations

BBB: Blood–brain barrier
bFGF: Basic fibroblast growth factor
BMEC: Brain microvascular endothelial cells
BSA: Bovine serum albumin
CNS: Central nervous system
CsA: Cyclosporin A
ECM: Extracellular matrix
HBVP: Human brain vascular pericytes
iPSC: Induced pluripotent stem cells
LY: Lucifer yellow
Papp: Apparent permeability
P-gp: P-glycoprotein
RA: Retinoic acid
R123: Rhodamine 123
TEER: Transendothelial electrical resistance
