# Supplementary material for: A Perfused In Vitro Human iPSC-Derived Blood–Brain Barrier Faithfully Mimics Transferrin Receptor-Mediated Transcytosis of Therapeutic Antibodies
Source: Cell Mol Neurobiol. 2023 Sep 12;43(8):4173–87. doi: 10.1007/s10571-023-01404-x (PMC10661771; doi:10.1007/s10571-023-01404-x)
Supplement: Supplementary file 1 — Supplementary file1 (DOCX 3380 KB) [file 10571_2023_1404_MOESM1_ESM.docx]

**A Perfused In Vitro Human iPSC-Derived Blood-Brain Barrier Faithfully Mimics Transferrin Receptor-Mediated Transcytosis of Therapeutic Antibodies**

Cellular and Molecular Neurobiology

Floriana Burgio^a^, Carine Gaiser^a^, Kevin Brady^b^, Viviana Gatta^c^, Reiner Class^b^, Ramona Schrage^c^, Laura Suter-Dick^a*^

^a^ University of Applied Sciences and Arts Northwestern Switzerland (FHNW), Muttenz, Switzerland

^b^ UCB Biopharma SRL, Development Sciences, Braine l’Alleud, Belgium

^c^ UCB Biopharma SRL, Neuroscience Therapeutic Area, Braine l’Alleud, Belgium

* Corresponding author

[laura.suterdick@fhnw.ch](mailto:laura.suterdick@fhnw.ch)

**
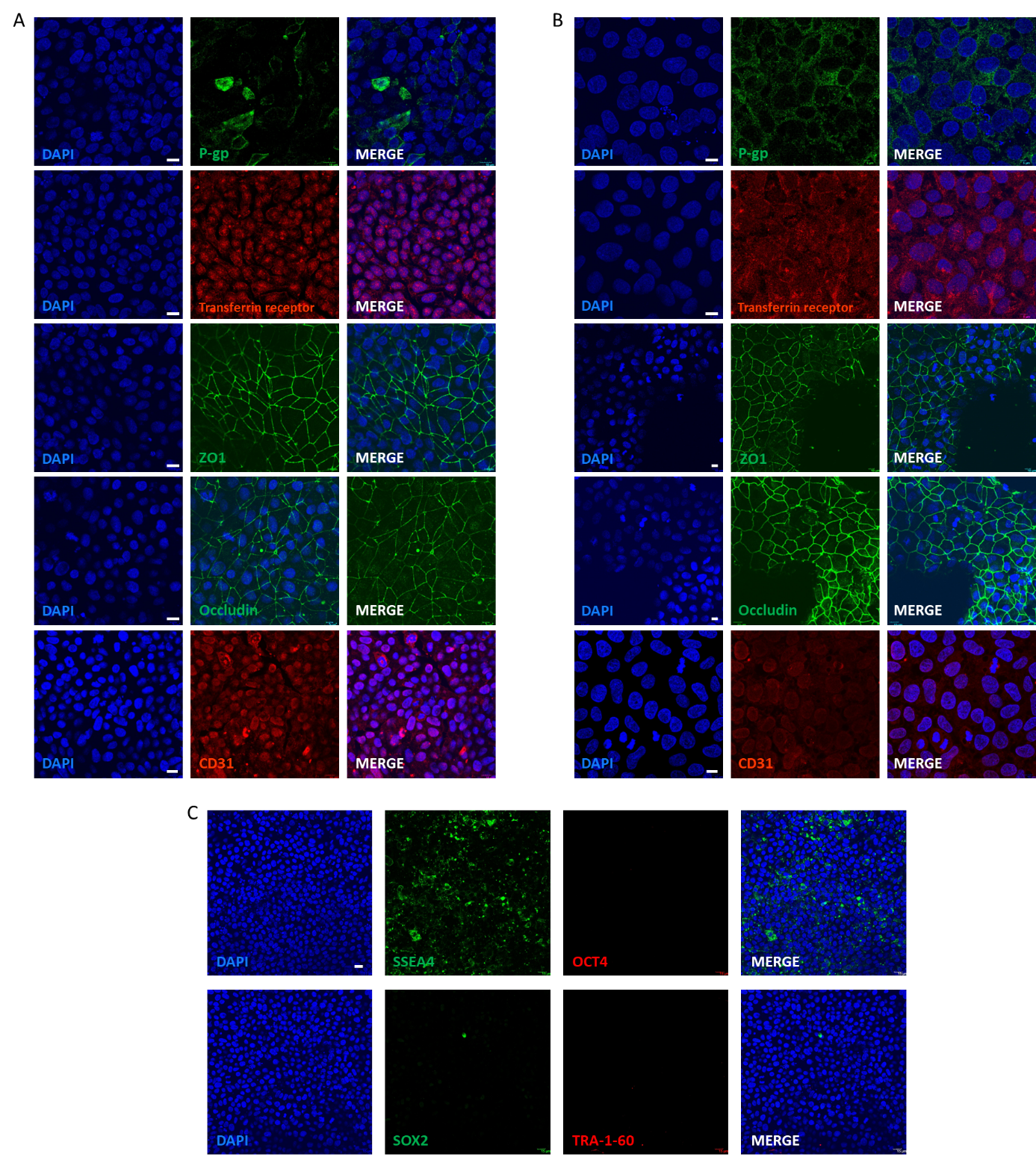
Fig. S1**

Characterization of iPSC-derived BMEC in 2D cell culture. The expression of BBB and endothelial markers was evaluated in iPSC-derived BMEC (**A**) and undifferentiated iPSC (**B**). Immunostaining of P-gp, ZO1 and Occludin (green) and transferrin receptor and CD31 (red). DAPI nuclear counter-staining (blue), scale bar: 10 µm. **C** Immunostaining of pluripotent stem cell markers on differentiated BMEC. OCT4 and TRA-1-60 (red) and SSEA4 and SOX2 (green), and DAPI nuclear counter-staining (blue), scale bar 15 µm

**
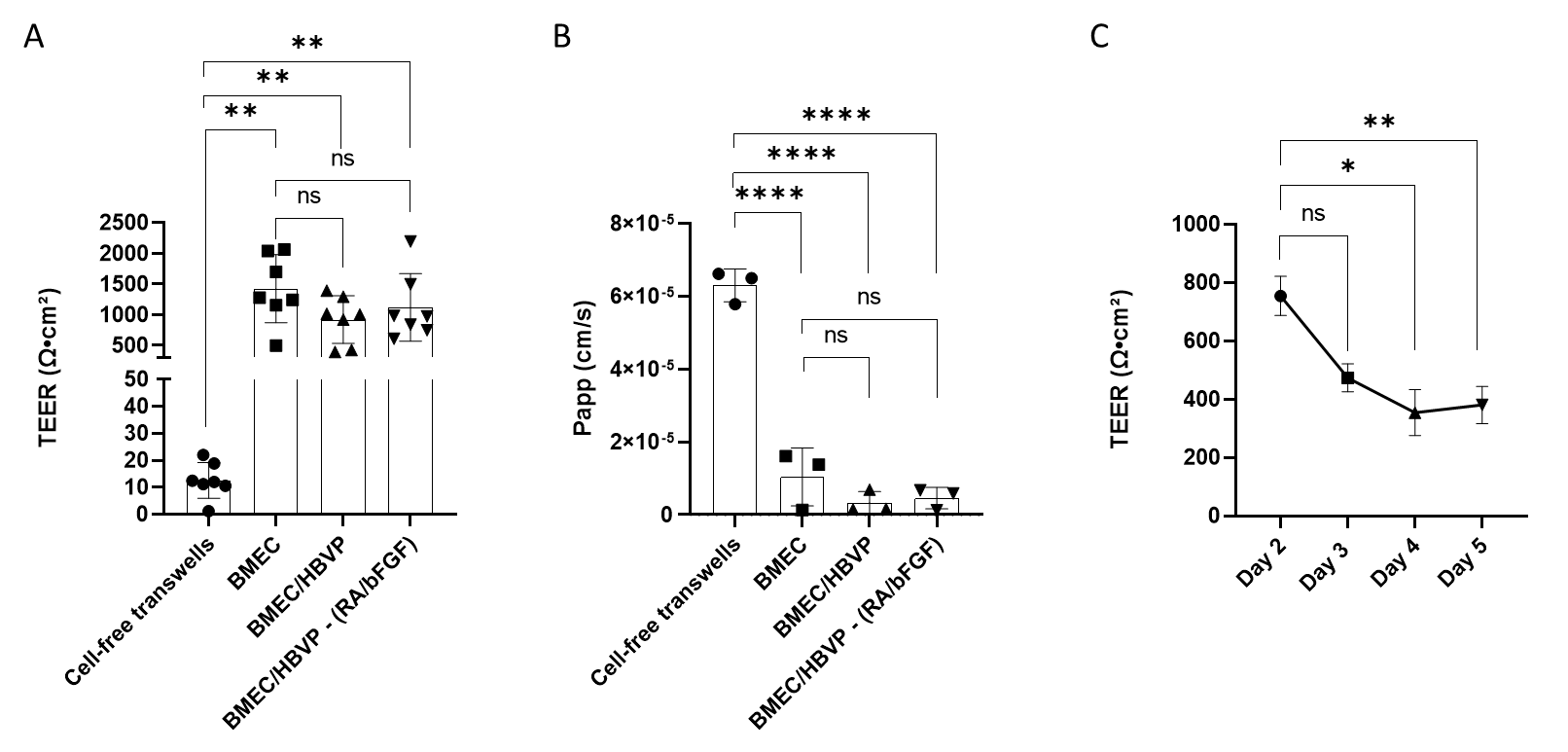
Fig. S2**

Characterization of the static-based BBB model. The influence of HBVP and the withdrawal of bFGF and RA on barrier formation were evaluated after 2 days of subculture by measuring: **A** TEER, expressed as mean ±SD (n=7 transwells per condition). The statistical analysis was performed using Welch ANOVA test, Dunnett’s T3 multiple comparisons test, **p < 0.01; **B** Papp of 100 µg/ml LY after 1 h incubation, expressed as mean ±SD (n=3 transwells per condition). The statistical analysis was performed using one-way ANOVA test, Dunnett’s T3 or Sidak's multiple comparisons test, ****p < 0.0001; **C** BMEC (100.000/0.33 cm^2^) were seeded on the apical side of the membrane and the integrity of the barrier was evaluated over 5 days by measuring TEER, expressed as mean ±SD (n=3 transwells per condition). The statistical analysis was performed using RM one-way ANOVA test with Geisser-Greenhouse correction, Dunnett’s T3 multiple comparisons test. *p < 0.05, **p < 0.01

**
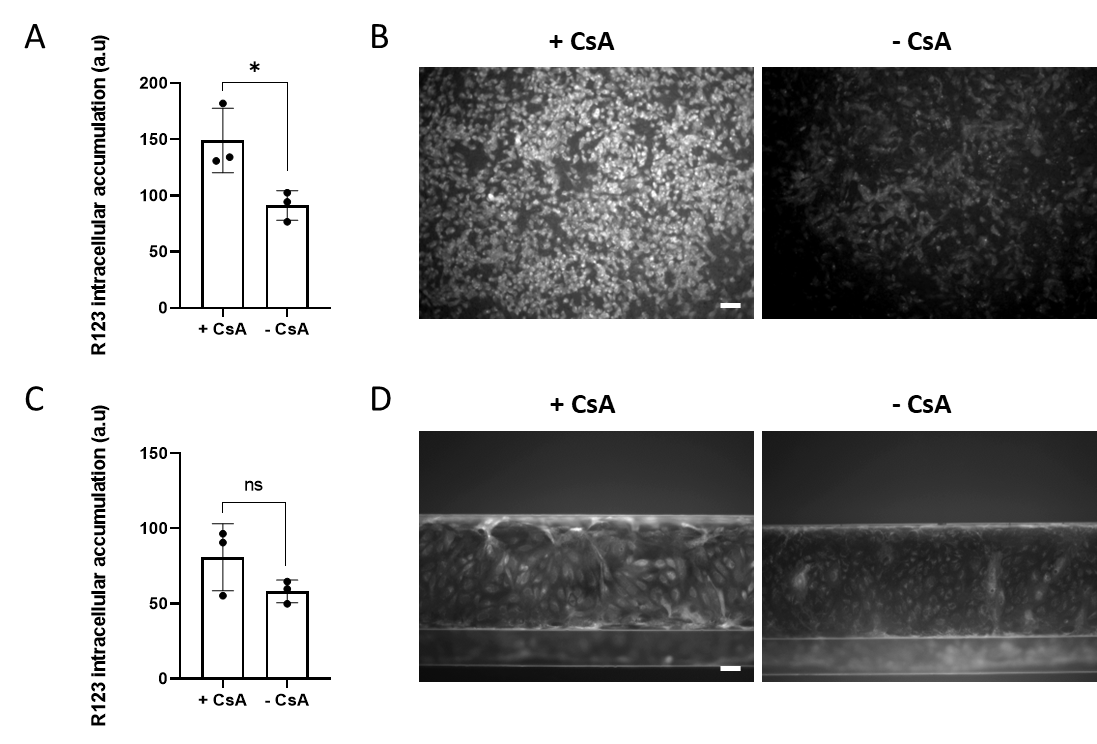
**

**Fig. S3**

Functionality of P-gp efflux pump. To show the activity of the P-gp pump, BMEC subcultured in 2D cell culture 96 wells plate (**A, B**) or 3D cell culture OrganoPlate® 3-lane (**C, D**) were incubated with 10 µM R123 with and without 10 µM CsA. Intracellular accumulation of R123 was evaluated by: **A, C** quantification, expressed as mean ± SD (n=3 wells or chips per condition). The statistical analysis was performed using unpaired t test. *p < 0.05; **B, D** fluorescence imaging, scale bar: 50 µm

**Fig. S4**

Transferrin receptor-mediated transcytosis. BMEC were incubated with a mixture of 500 µg/ml unlabeled transferrin and BSA for 2 h. The concentration of transferrin and BSA in the bottom channel was quantified by ELISA and Papp was calculated accordingly, expressed as median with interquartile range (n=4 chips per condition). The statistical analysis was performed using Wilcoxon matched-pairs signed rank test when comparison refers to BMEC or paired t test when comparison refers to cell-free chips, ***p < 0.001

# Results of statistical analysis

**Fig. 2C:**

Results of one-tail two-sample F-test for variances

|  | 1 h | | 2 h | | 3 h | |
| --- | --- | --- | --- | --- | --- | --- |
| F | 0.0978 | | 0.1328 | | 0.5795 | |
| P | 0.0018 | | 0.0049 | | 0.2286 | |
| Df | Cell-free chips  8 | BMEC  8 | Cell-free chips  8 | BMEC  8 | Cell-free chips  8 | BMEC  8 |

Results of normality test (Shapiro-Wilk test)

|  | 1 h | | 3 h | | 6 h | |
| --- | --- | --- | --- | --- | --- | --- |
|  | W | P | W | P | W | P |
| Cell-free chips | 0.8962 | 0.2307 | 0.9616 | 0.8151 | 0.8064 | 0.0242 |
| BMEC | 0.5295 | <0.0001 | 0.6359 | 0.0003 | 0.7127 | 0.0020 |

Results of two-tail Mann-Whitney test: independently on each time point, P<0.0001.

**Fig. 2E:**

Results of one-tail two-sample F-test for variances

|  | 1 h | | 2 h | | 3 h | | 4 h | |
| --- | --- | --- | --- | --- | --- | --- | --- | --- |
| F | 617.046 | | 372.179 | | 363.997 | | 340.4842 | |
| P | 7.8e-06 | | 2.4e-05 | | 2.2e-05 | | 2.6e-05 | |
| Df | Cell-free chips  4 | BMEC  4 | Cell-free chips  3 | BMEC  4 | Cell-free chips  4 | BMEC  4 | Cell-free chips  4 | BMEC  4 |

Results of normality test (Shapiro-Wilk test)

|  | 1 h | | 2 h | | 3 h | | 4h | |
| --- | --- | --- | --- | --- | --- | --- | --- | --- |
|  | W | P | W | P | W | P | W | P |
| Cell-free chips | 0.7407 | 0.0244 | 0.7479 | 0.0285 | 0.7779 | 0.0528 | 0.7898 | 0.0668 |
| BMEC | 0.5522 | 0.0001 | 0.5522 | 0.0001 | 0.5522 | 0.0001 | 0.5522 | 0.0001 |

Results of two-tail Mann-Whitney test: independently on each time point, P=0.0079.

**Fig. 4A:**

Results of Brown-Forsythe test for variances (Cells-free chips, BMEC, BMEC + Mannitol)

|  | 15 min | 30 min | 1 h | 3 h | 6 h |
| --- | --- | --- | --- | --- | --- |
| P | 0.0020 | 0.0001 | 0.0390 | 0.0904 | 0.2013 |

Results of normality test (Shapiro-Wilk test)

|  | 15 min | | 30 min | | 1 h | | 3 h | | 6 h | |
| --- | --- | --- | --- | --- | --- | --- | --- | --- | --- | --- |
|  | W | P | W | P | W | P | W | P | W | P |
| Cell-free chips | 0.8930 | 0.2492 | 0.8633 | 0.1295 | 0.8653 | 0.1356 | 0.8400 | 0.0753 | 0.8938 | 0.2538 |
| BMEC | / | / | 0.5108 | <0.0001 | 0.6074 | 0.0002 | 0.7077 | 0.0028 | 0.7530 | 0.0088 |
| BMEC + Mannitol | 0.8217 | 0.0486 | 0.9207 | 0.4354 | 0.9182 | 0.4156 | 0.9330 | 0.5434 | 0.9714 | 0.9087 |

Results of Kruskal-Wallis test (Cells-free chips, BMEC, BMEC + Mannitol): independently on each time point, P<0.0001.

Dunn's multiple comparisons test:

|  | 15 min | 30 min | 1 h | 3 h | 6 h |
| --- | --- | --- | --- | --- | --- |
| Cell-free chips vs. BMEC | P<0.0001 | P<0.0001 | P<0.0001 | P<0.0001 | P<0.0001 |
| Cell-free chips vs. BMEC + Mannitol | P=0.1951 | P=0.0452 | P=0.0451 | P=0.0472 | P=0.0473 |
| BMEC vs. BMEC + Mannitol | P=0.0171 | P=0.0452 | P=0.0451 | P=0.0472 | P=0.0473 |

**Fig. 5C:**

Results of one-tail two-sample F-test for variances

| F | 6.4797 | |
| --- | --- | --- |
| P | 0.0796 | |
| Df | Ratio transferrin  3 | Ratio BSA  3 |

Results of normality test (Shapiro-Wilk test)

|  | W | P |
| --- | --- | --- |
| Transferrin | 0.9422 | 0.6679 |
| BSA | 0.6298 | 0.0012 |

Results of two-tail Mann-Whitney test: P=0.0286.

**Fig. 6B:**

Results of one-tail two-sample F-test for variances

|  | Cell-free chips | | BMEC | |
| --- | --- | --- | --- | --- |
| F | 0.0838 | | 34.985 | |
| P | 0.0083 | | 0.0007 | |
| Df | MEM-189  5 | Control antibody  5 | MEM-189  5 | Control antibody  5 |

Results of normality test (Shapiro-Wilk test)

|  | Cell-free chips | | BMEC | |
| --- | --- | --- | --- | --- |
|  | W | P | W | P |
| MEM-189 | 0.9292 | 0.5737 | 0.8138 | 0.0708 |
| Control antiibody | 0.9129 | 0.4558 | 0.6902 | 0.0049 |

Results of two-tail paired t test for Cell-free chips, MEM-189 vs. Control antibody: P=0.7872, t=0.2848, df=5.

Results of two-tail Wilcoxon matched-pairs signed rank test for BMEC, MEM-189 vs. Control antibody: P=0.0313.

**Fig. S2A:**

Results of Bartlett’s test for variances: P<0.0001.

Results of normality test (Shapiro-Wilk test)

|  | W | P |
| --- | --- | --- |
| Cell-free inserts | 0.9290 | 0.5423 |
| BMEC | 0.9259 | 0.5169 |
| BMEC/HBVP | 0.8978 | 0.3178 |
| BMEC/HBVP - (RA/bFGF) | 0.8391 | 0.0975 |

Results of Welch’s ANOVA test: W (DFn, DFd)= 32.87 (3.000, 10.00), P<0.0001.

Dunnett's T3 multiple comparisons test:

| Cell-free inserts vs. BMEC | P=0.0015 |
| --- | --- |
| Cell-free inserts vs. BMEC/HBVP | P=0.0023 |
| Cell-free inserts vs. BMEC/HBVP - (RA/bFGF) | P=0.0050 |
| BMEC vs. BMEC/HBVP | P=0.1398 |
| BMEC vs. BMEC/HBVP - (RA/bFGF) | P=0.5307 |

**Fig. S2B:**

Results of Brown-Forsythe test for variances: P=0.738.

Results of normality test (Shapiro-Wilk test)

|  | W | P |
| --- | --- | --- |
| Cell-free inserts | 0.8586 | 0.2637 |
| BMEC | 0.8659 | 0.2839 |
| BMEC/HBVP | 0.7678 | 0.0398 |
| BMEC/HBVP - (RA/bFGF) | 0.8598 | 0.2669 |

Results are considered normally distributed since the groups of Cell-free chips, BMEC and BMEC/HBVP - (RA/bFGF) are normally distributed and the group BMEC/HBVP has p=0.0398 close to 0.05.

Results of one-way ANOVA test: F=96.63, P<0.0001, R squared=0.9731.

Dunnett's T3 multiple comparisons test:

| Cell-free inserts vs. BMEC | P<0.0001 |
| --- | --- |
| Cell-free inserts vs. BMEC/HBVP | P<0.0001 |
| Cell-free inserts vs. BMEC/HBVP - (RA/bFGF) | P<0.0001 |

Sidak's multiple comparisons test:

| BMEC vs. BMEC/HBVP | P=0.2341 |
| --- | --- |
| BMEC vs. BMEC/HBVP - (RA/bFGF) | P=0.3532 |

**Fig. S2C:**

Results of Brown-Forsythe test for variances: P=0.9480.

Results of normality test (Shapiro-Wilk test)

|  | W | P |
| --- | --- | --- |
| Day 2 | 0.9327 | 0.4990 |
| Day 3 | 0.9655 | 0.6433 |
| Day 4 | 0.9998 | 0.9741 |
| Day 5 | 0.9120 | 0.4249 |

Results of RM one-way ANOVA test: No sphericity assumed, F=61.51, P=0.0039, Geisser-Greenhouse’s epsilon=0.5010, R squared=0.9865.

Dunnett's T3 multiple comparisons test:

| Day 2 vs. Day 3 | P=0.0564 |
| --- | --- |
| Day 2 vs. Day 4 | P=0.0124 |
| Day 2 vs. Day 5 | P=0.0046 |

**Fig. S3A and Fig. S3C:**

Results of one-tail two-sample F-test for variances

|  | Fig.S3A | | Fig.S3C | |
| --- | --- | --- | --- | --- |
| F | 8.7816 | | 4.7068 | |
| P | 0.1022 | | 0.1752 | |
| Df | + CsA  2 | - CsA  2 | + CsA  2 | - CsA  2 |

Results of normality test (Shapiro-Wilk test)

|  | Fig.S3A | | Fig.S3C | |
| --- | --- | --- | --- | --- |
|  | W | P | W | P |
| + CsA | 0.8547 | 0.2530 | 0.7979 | 0.1094 |
| - CsA | 0.9633 | 0.6317 | 0.9573 | 0.6027 |

Fig. S3A Results of two-tail unpaired t test: P=0.1701, t=1.671, df=4.

Fig. S3C Results of two-tail unpaired t test: P=0.0336, t=3.178, df=4.

**Fig. S4:**

Results of one-tail two-sample F-test for variances

|  | Cell-free chips | | BMEC | |
| --- | --- | --- | --- | --- |
| F | 0.0161 | | 0.3846 | |
| P | 0.0034 | | 0.2267 | |
| Df | Transferrin  3 | BSA  3 | Transferrin  3 | BSA  3 |

Results of normality test (Shapiro-Wilk test)

|  | Cell-free chips | | BMEC | |
| --- | --- | --- | --- | --- |
|  | W | P | W | P |
| Transferrin | 0.9516 | 0.7261 | 0.9546 | 0.7452 |
| BSA | 0.9021 | 0.4414 | 0.6298 | 0.0012 |

Results of two-tail paired t test for Cell-free chips, Transferrin vs. BSA: P=0.0009, t=13.40, df=3.

Results of two-tail Wilcoxon matched-pairs signed rank test for BMEC, Transferrin vs. BSA: P=0.1250.

# Antibody specificity

Validation data of the following primary antibodies are provided by the supplier as follows:

- GLUT1 Antibody (MA5-31960; RRDI:AB_2809254): Antibody specificity was demonstrated by siRNA mediated knockdown of target protein.

- INSR Antibody (MA5-13783; RRDI:AB_10985120): Antibody specificity was demonstrated by detection of differential basal expression of the target across Reh and Jurkat owing to their inherent genetic constitution.

- LDLR Antibody (PA5-115504; RRDI:AB_2900140): Antibody specificity was demonstrated in Western blot analysis performed in HepG2 cells where the target protein was treated with and without blocking peptide before incubation with the polyclonal antibody.

- Occludin Antibody (33-1500; RRDI:AB_2533101): Antibody specificity was demonstrated by CRISPR-Cas9 mediated knockout of target protein.

- PAX6 Antibody (42-6600; RRDI:AB_2533534): Antibody specificity was demonstrated by siRNA mediated knockdown of target protein.

- P-Glycoprotein Antibody (MA5-13854; RRDI:AB_10979045): Antibody specificity was demonstrated by CRISPR-Cas9 mediated knockout of target protein.

- SOX17 Antibody (MA5-24885; RRDI:AB_2725396): Antibody specificity was demonstrated by detection of differential basal expression of the target across cell models owing to their inherent genetic constitution.

- VWF Antibody (MA5-14029; RRDI:AB_11001165): Antibody specificity was demonstrated by detection of differential basal expression of the target across cell models owing to their inherent genetic constitution.

- ZO-1 Antibody (33-9100; RRDI:AB_2533147): Antibody specificity was demonstrated by siRNA mediated knockdown of target protein.

- Claudin-5 Antibody (ab15106; RRDI:AB_301652): antibody specificity was demonstrated in IHC-P application by looking at cells that either do or do not express the target protein within the same tissue.

- CD31 Antibody (ab28364; RRDI:AB_726362): antibody specificity was demonstrated in IHC-P application by looking at cells that either do or do not express the target protein within the same tissue.

Based on the datasheet, Claudin-5 Antibody is unsuitable for: ICC/IF. However, its application in IF has been reported in the literature: PMID: 34713920, DOI: 10.1002/glia.24109, PMID: 36056066, DOI: 10.1038/s41598-022-18964-5.

- Transferrin receptor Antibody (ab84036; RRDI:AB_10673794): antibody specificity was demonstrated in ICC application by looking at cells that either do or do not express the target protein within the same tissue.

- Brachyury Antibody (14-9770-82; RRDI:AB_2573016): antibody specificity was demonstrated in ICC and IHC applications by looking at the localization within the cell or tissue samples that express the target protein. Moreover, antibody specificity was demonstrated in Western blot analysis by showing that the antibody recognizes the protein based on a molecular weight within a sample that expresses the protein.
